# Blockade of NF-κB nuclear translocation results in the inhibition of the invasiveness of human gastric cancer cells

**DOI:** 10.3892/ol.2013.1390

**Published:** 2013-06-11

**Authors:** ZHI-MIN LI, YU-WEI PU, BAO-SONG ZHU

**Affiliations:** 1 Department of General Surgery, The First Wujiang Affiliated Hospital, Nantong University, Wujiang, Jiangsu 215200, P.R. China; 2 Department of General Surgery, The Second Affiliated Hospital, Soochow University, Suzhou, Jiangsu 215004, P.R. China

**Keywords:** NF-κB p65, SN50, gastric cancer, invasiveness

## Abstract

The aim of the present study was to investigate the effect of the nuclear factor-κB (NF-κB) p65 inhibitor, SN50, on the invasiveness and mechanisms of SGC7901 human gastric carcinoma cell xenografts in nude mice. Nude mice were randomly divided into model control and SN50 treatment groups. On days 5, 10 and 15 following treatment, the tumor samples were observed and a selection of parameters were recorded, including the level of tumor growth inhibition, the pathological changes in the tumor specimens, the expression levels of matrix metalloproteinase-9 (MMP-9), proliferating cell nuclear antigen (PCNA), tissue inhibitor of metalloproteinases type-1 (TIMP-1) and vascular endothelial growth factor (VEGF) and the apoptosis indices in the tumor samples. The results demonstrated that treating the tumor with SN50 for 5, 10 and 15 days inhibited carcinoma growth in comparison with the control group. Hematoxylin and eosin (HE) staining indicated that the level of inhibition increased progressively, in correlation with apoptosis. The expression of the MMP-9, PCNA and VEGF proteins was observed to be downregulated, while that of the TIMP-1 protein was shown to be upregulated, using immunohistochemical staining. In conclusion, the NF-κB p65 inhibitor, SN50, inhibited the invasiveness of the gastric cancer cells by downregulating the protein expression of MMP-9, PCNA and VEGF and upregulating the protein expression of TIMP-1. It was further suggested that SN50 may be a molecular target of anti-invasion therapy for gastric cancer, and that the inhibition of the NF-κB p65 signaling pathway may be considered as a potential strategy for treating gastric cancer.

## Introduction

Gastric cancer is the fourth most frequently diagnosed malignancy worldwide, accounting for 12% of all cancer-related mortalities. In Asia and parts of South America in particular, gastric cancer is the most common epithelial malignancy and is a leading cause of cancer-related mortality (1,2).

Proliferation, invasiveness and metastasis are the predominant biological characteristics of a malignant tumor, and are closely correlated with factors such as the movement of the tumor cells, apoptosis and the expression of metastasis-associated genes. Matrix metalloproteinase-9 (MMP-9), tissue inhibitor of metalloproteinases type-1 (TIMP-1) (3) and vascular endothelial growth factor (VEGF) are important angiogenic factors that have a higher expression level in tumor tissues. These factors induce angiogenesis in the tumor and are important in the metastasis, invasion and prognosis of gastric cancer (4–8). Proliferating cell nuclear antigen (PCNA) (9) is a protein that is widely expressed in the S phase of the cell cycle, and the levels of PCNA reflect the proliferative activity of the tumor cells. Studies have demonstrated that the PCNA proliferation index increases in correlation with the histological grading and staging of the malignant degree of progress (10,11). Levels of PCNA are considered to be a reliable indicator of cell proliferation, due to the correlation between PCNA levels and the proliferative activity of tumor cells (10,12). However, the underlying mechanisms behind this remain unclear

Nuclear factor-κB (NF-κB) has been linked to the control of cell growth and oncogenesis. The mechanisms of NF-κB in cancer appear to be complex, but are likely to involve the ability of this transcription factor to control programmed cell death (PCD) and cell cycle progression, as well as cell differentiation, angiogenesis and cell migration. It has been demonstrated that NF-κB is activated in cancer cells by several types of chemotherapy and by radiation, and that in a number of instances this response inhibits the radiotherapy and chemotherapy-induced death of the cancer cells (13). Therefore, the inhibition of NF-κB p65 is under investigation as a potentially useful approach in the treatment of cancer. However, the detailed mechanisms are poorly understood. The present study investigated the effects of the nuclear import inhibitor, SN50, on the growth and invasiveness of implanted SGC7901 cell tumors in nude mice, and the relative mechanism involved.

## Materials and methods

### Materials

SGC7901 cells and female Balb/c nude mice (age, 4 weeks; weight, 16–18 g) were purchased from the Chinese Academy of Sciences (Shanghai, China). RPMI-1640 medium was obtained from Gibco (Rockville, MD, USA), and fetal bovine serum (FBS) was provided by Hangzhou Sijiqing Biological Engineering Material Co., Ltd. (Hangzhou, China). Anti-MMP-9, -PCNA and -TIMP-1 monoclonal antibodies and PCNA, TIMP-1 and VEGF were purchased from Santa Cruz Biotechnology, Inc. (Santa Cruz, CA, USA). The streptavidin-peroxidase kit was purchased from Fuzhou Maixin Biotechnology Development Co., Ltd. (Fuzhou, China). This study was approved by the Ethics Committee of The Second Affiliated Hospital, Soochow University, Suzhou, China.

### Drug preparation

The SN50 (Biomol, Plymouth Meeting, PA, USA) was diluted in sterilized distilled water to create a stock solution that was stored in accordance with the manufacturer’s instructions. The final concentration of the SN50 solution used was 18 *μ*mol/l. This concentration was selected on the basis of our previous experiments on implanted human gastric cancer SGC7901 cells in nude mice (14).

### Cell culture

The SGC7901 cells were maintained in RPMI-1640 medium (Gibco) containing 10% heat-inactivated FBS (Hangzhou Sijiqing Biological Engineering Material Co., Ltd.) and 0.03% L-glutamine (Sigma, St. Louis, MO, USA), and incubated in a 5% CO_2_ atmosphere at 37°C. Cells in the mid-log phase were used in the experiments.

### Level of inhibition of tumor growth

A transplanted tumor model was established by injecting human SGC7901 cells (1x10^9^ ml) into the subcutaneous tissue of the armpit of nude mice. Ten days later, the 25 nude mice were randomly divided into five groups as follows: i) control, ii) phosphate-buffered saline (PBS), iii) 5 days after SN50 treatment, iv) 10 days after SN50 treatment and v) 15 days after SN50 treatment. Then, 0.2 ml normal saline solution, 1.5 mg/kg PBS or SN50 (18 *μ*mol/l) were directly injected adjacent to the tumor three times, at 2-day intervals. Changes in tumor volume were calculated using the following formula: V = (π/6) x abc, where a is the length of the tumor, b is the width of the tumor and c is the depth of the tumor). These changes were measured at 5, 10 and 15 days after the SN50 treatment. The level of inhibition of tumor growth in each group was calculated as follows: level of inhibition of tumor growth = [C(V1-V0) − T(V1-V0)] / C(V1-V0) x 100, where C is the control group, T is the treatment group, V1 is the volume prior to treatment (mm^3^) and V0 is the volume following treatment (mm^3^).

### Hematoxylin and eosin (HE), and immunohistochemical staining

Tumor specimens were taken from areas adjacent to the margins of the tumors and from central areas. The specimens were formalin-fixed, paraffin-embedded and pathologically diagnosed as gastric carcinoma. The specimens were then evaluated by HE staining for a conventional histological assessment. The histological characteristics were reviewed by two pathologists.

The tumor samples were cut into 4-*μ*m thick slices and fixed in acetone. Following washing in PBS, the slices were incubated in 0.3% H_2_O_2_ solution at room temperature for 5 min. The slices were then incubated with anti-TIMP-1, -MMP-9, -PCNA or -VEGF monoclonal antibodies at a 1:300 dilution at 4°C overnight. Following further washing in PBS, a second antibody, biotinylated anti-rat immunoglobulin G (IgG), was added and the cells were incubated at room temperature for 1 h. The cells were then washed again in PBS, avidin-biotin complex (ABC) compound was added and the cells were subsequently incubated at room temperature for 10 min. 3,3′-Diaminobenzidine (DAB) was used as the chromogen. Following incubation, the brown color signifying the presence of antigens binding to antibodies was detected by light microscopy. The controls were prepared in the same manner as the experimental group, with the exception of the incubation with the primary antibody. The positive rate (PR) of protein expression was calculated as follows: PR = (number of positive cells / total number of cells) x 100.

### Immunohistochemical assessment

The cytoplasm of the cells containing MMP-9, PCNA, TIMP-1 and VEGF was brown in appearance. The immunohistochemical staining was independently evaluated by two pathologists, who were blinded to the clinical data. In total, 200 cells were selected under the microscope to evaluate the stained cell number against the total cell number in the field. Based on the positive cell number, the criteria were set as follows: negative −, <10% positive cells; +, 11–50% positive cells; ++, 51–75% positive cells; and +++, >75% positive cells. The staining results for the presence of MMP-9, PCNA, TIMP-1 and VEGF were classified into negative (staining of ≤10% of cells) or positive (staining of >10% of cells) results.

### Statistical analysis

Data are presented as mean ± standard deviation. The statistical analysis was carried out using an ANOVA, followed by Dunnett’s t-test. P<0.05 was considered to indicate a statistically significant difference.

## Results

### Effect of SN50 on tumor growth

SGC7901 cells (1x10^9^) were injected subcutaneously into the armpits of nude mice. Within 1 week, visible tumors had developed at the injection sites. To determine the therapeutic effectiveness of the SN50, intratumoral injections of SN50 were administered once the volume of the implanted tumor reached 20 mm^3^, and were repeated every 2 days three times in total. As shown in Table I, SN50 suppressed tumor growth compared with the control group (P<0.01). No gross adverse effects, e.g. the loss of body weight, were observed during the experimental period. Furthermore, SN50 inhibited the proliferation of the implanted human gastric cancer SGC7901 cells in the nude mice, in a time-dependent fashion. The level of inhibition of the tumors were 8.2±2.1, 19.7±1.6 and 28.3±2.6% following treatment with the SN50 for 5, 10 and 15 days, respectively (Table I).

### SN50 inhibits cell proliferation and induces cell death of transplanted SGC7901 tumor cells

**Figure 1.**
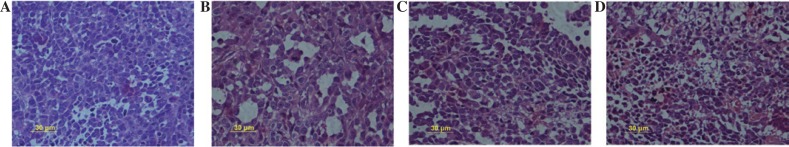
SN50-induced cell death of transplanted SGC7901 tumor cells. Pathological changes in the SGC7901 tumor cells in (A) the model control and (B–D) experimental groups. (B) 5, (C) 10 and (D) 15 days after treatment with SN50. Hematoxylin and eosin (HE) staining; magnification, x200.

**Figure 2.**
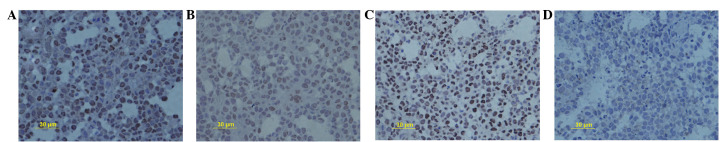
SN50 inhibits proliferating cell nuclear antigen (PCNA) protein expression. Pathological changes in the SGC7901 tumor cells in (A) the model control and (B–D) experimental groups. (B) 5, (C) 10 and (D) 15 days after treatment with SN50. Hematoxylin and eosin (HE) staining; magnification, x200.

Treatment of the SGC7901 cells with SN50 for 5, 10 and 15 days produced intensive HE staining, indicating apoptosis. An increase in cell death was observed in correlation with the treatment period of the tumors (Fig. 1). Five days after the SN50 treatment, the level of inhibition was 13.5±2.3%. The level of inhibition increased as the experiment progressed, reaching 25.6±3.1% on day 10 and 32.9±2.7% on day 15 following the SN50 treatment. The results indicated that 18 *μ*ol/l SN50 induced cell death (Fig. 1). It was observed that SN50 inhibited cell proliferation by decreasing PCNA protein expression from 59.2±2.4% in the control group to 46.3±1.2, 37.5±1.9 and 28.3±1.6% in the experimental groups, following treatment with 18 *μ*mol/l SN50 for 5, 10 and 15 days, respectively (Fig. 2A–D, respectively).

### SN50 inhibits the expression of MMP-9 protein

**Figure 3.**
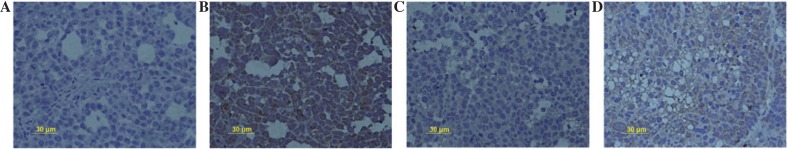
SN50 inhibits matrix metalloproteinase-9 (MMP-9) protein expression. Pathological changes in the SGC7901 tumor cells in (A) the model control and (B–D) experimental groups. (B) 5, (C) 10 and (D) 15 days after treatment with SN50. Hematoxylin and eosin (HE) staining; magnification, x200.

Positive staining for MMP-9 protein was distributed in the cell membrane and cytoplasm. The PR for MMP-9 protein expression decreased from 46.2±2.1% in the control group to 33.7±1.3, 21.6±0.7 and 9.3±1.2% in the experimental groups, following treatment with 18 *μ*mol/l SN50 for 5, 10 and 15 days, respectively (Fig. 3A–D, respectively). Significant differences were observed in MMP-9 protein expression between the 18 *μ*mol/l SN50 group and the control group at every time-point (P<0.05).

### SN50 upregulates the expression of TIMP-1 protein

**Figure 4.**
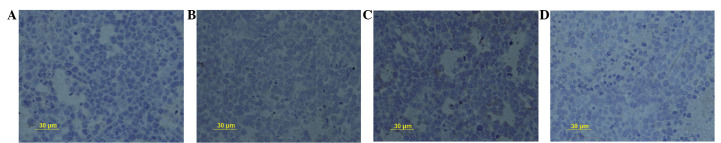
SN50 increases tissue inhibitor of metalloproteinases type-1 (TIMP-1) protein expression. Pathological changes in the SGC7901 tumor cells in (A) the model control and (B–D) experimental groups. (B) 5, (C) 10 and (D) 15 days after treatment with SN50. Hematoxylin and eosin (HE) staining; magnification, x200.

Positive staining was observed for TIMP-1 protein in the cytoplasm in the healthy mucosa adjacent to the cancer cells. The PR for TIMP-1 protein was upregulated from 23.2±2.1% in the control group to 35.4±2.0, 47.9±1.7 and 31.9±2.3% following treatment with SN50 for 5, 10 and 15 days, respectively (Fig. 4A–D, respectively). Significant differences were observed in TIMP-1 protein expression between the 18 *μ*mol/l SN50 group and the control group at every time-point (P<0.05).

### SN50 inhibits the expression of VEGF

**Figure 5.**
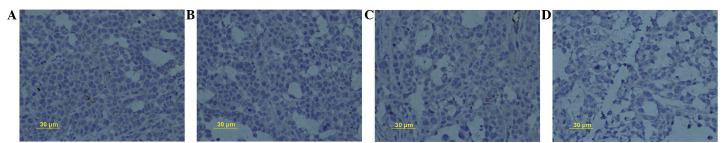
SN50 decreases vascular endothelial growth factor (VEGF) protein expression. Pathological changes in the SGC7901 tumor cells in (A) the model control and (B–D) experimental groups, (B) 5, (C) 10 and (D) 15 days after treatment with SN50. Hematoxylin and eosin (HE) staining; magnification, x200.

The PR for VEGF protein expression indicated that the expression of VEGF was downregulated from 46.2±2.3% in the control group to 28.7±1.2, 16.3±1.4 and 12.1±2.6% in the experimental groups, following treatment with 18 *μ*mol/l SN50 for 5, 10 and 15 days, respectively (Figs. 5A–D, respectively). A significant difference in positive expression was observed between the 18 *μ*mol/l SN50 group and the control group at every time-point (P<0.05).

## Discussion

At present, there are relatively few chemotherapeutic drugs that are effective in the treatment of human gastric carcinoma (15). As a result, there is an increasing body of interest in the use of drugs that prevent the invasion of cancer cells. NF-κB signaling pathways are important in a variety of physiological and pathological processes. One of the functions of NF-κB is the promotion of cell survival through the induction of target genes, whose products inhibit components of the apoptotic machinery in normal and cancerous cells. Regardless of the mechanism, numerous cancer cells, of either epithelial or hematopoietic origin, use NF-κB to achieve a resistance to anticancer drugs, radiation and death cytokines. Hence, the inhibition of NF-κB activation offers a future potential strategy for the treatment of different malignancies, and may induce cell death in gastric cancer SGC7901 cells (16–18).

Tumor metastasis involves a series of complex processes in which numerous gene products feature. MMPs, which are important in the breakdown of the extracellular matrix (ECM), are overexpressed in malignant tumors and have been demonstrated to contribute to tumor proliferation, invasion and metastasis (19). Among the MMPs, MMP-9 has a close association with tumor metastasis, and is considered, in particular, to be an important factor in facilitating invasion and metastasis in gastric carcinoma (20–22). TIMPs (TIMP-1, -2, -3 and -4) have been demonstrated to be the key regulators of MMP activity and ECM degradation (23). The MMP inhibitors, TIMP-1 and TIMP-2, have been implicated in several tumorigenic processes, including the development, invasion and metastasis of bronchial cancer (24–28). Additionally, the level of PCNA reflects the proliferative activity of the tumor cells, and is considered to be a reliable indicator of cell proliferation.

VEGF acts to accelerate the formation of blood vessels, and also plays a vital role in tumor-associated microvascular invasion (29–31). It has been demonstrated that tumor metastasis is accelerated by the presence of VEGF, which is highly expressed in gastric carcinomas. VEGF may therefore be used as a marker of a poor prognosis in gastric carcinoma patients (32–34).

In the present study, there was a significant difference in the protein expression of MMP-9, VEGF, TIMP-1 and PCNA between the experimental treatment and control groups, respectively (P<0.05), indicating that SN50 may have inhibited the expression of MMP-9, PCNA and VEGF and upregulated the expression of TIMP-1. It was also demonstrated that SN50 inhibited cell proliferation and induced apoptosis in the implanted human SGC7901 gastric cancer cells, thus demonstrating the cytotoxic effects of SN50. *In vitro* invasion assays and *in vivo* nude mice assays suggested that SN50 had the potential to inhibit the invasion and metastasis of gastric cancer. This may have been due to the decrease in the protein expression of MMP-9, PCNA and VEGF, and the increase in the TIMP-1 protein expression induced by SN50, in combination with the cytotoxicity of SN50 towards the tumor cells. Furthermore, no gross adverse effects, e.g. the loss of body weight, were observed during the experimental period. These results indicate that the inhibition of NF-KB p65 is a potent and safe strategy for treating gastric cancer, and thus suggest that the development of SN50-based therapeutics may be an approach for the next generation of gastric cancer treatment.

## Figures and Tables

**Table I.** Inhibitory effect of SN50 on implanted tumors in nude mice.

Group	Number of animals		Level of inhibition (%)
	Start	End	
Control	5	5	0
PBS	5	5	1.65±0.12
SN50	15	15	
5 days	5	5	8.2±2.1
10 days	5	5	19.7±1.6^a^
15 days	5	5	28.3±2.6^a^

a P<0.05 vs. control group. Data are presented as mean ± standard deviation. PBS, phosphate-buffered saline.
